# The Roles of Mental Construal Level Theory in the Promotion of University Students' Pro-environmental Behaviors

**DOI:** 10.3389/fpsyg.2021.735837

**Published:** 2021-10-28

**Authors:** Yanhui Jia, Jie Tian, Haiyue Liu

**Affiliations:** ^1^Research Centre of the Economy of the Upper Reaches of the Yangtze River, Chongqing Technology and Business University, Chongqing, China; ^2^Business School, Sichuan University, Chengdu, China

**Keywords:** climate change adaptation, climate change mitigation, risk compensation behavior, construal level theory, pro-environmental behavior (PEB)

## Abstract

Although green technological innovation is designed to combat climate change, recent research suggests that increased attention to technological innovations might decrease climate change risk perception and reduce pro-environmental behaviors due to the feeling of being assured, which is referred to as risk compensation behavior. Although there has been a growing interest in reducing the risk compensation effect related to climate change, the academic literature in this area is very limited. In this study, we propose a psychological intervention to mitigate a sample of university students' (*N* = 1,500) irrational response to green technological innovation so as to promote their pro-environmental behaviors. Our experiments identify students' mental construal level as an important psychological factor that, when combined with a proper message framing strategy of introducing new green technologies, can remedy their irrational response to new green technologies. Our findings suggest that highlighting the new technology as playing a preventive/promotional role related to climate change can mitigate risk compensation behavior and eventually promote students' pro-environmental behaviors when they are at a high/low mental construal level.

## Introduction

Most simulations suggest that the change in the global surface temperature between 1,850 and the end of the twenty-first century is likely to exceed 1.5°C. The World Meteorological Organization (WMO) says that if the current warming trend continues, temperatures could rise 3–5°C by the end of this century. Temperature rises of 2°C have long been regarded as the gateway to dangerous warming. The Intergovernmental Panel on Climate Change report of 2014 states that a 70% reduction in anthropogenic GHG emissions between 2010 and 2050 is needed to limit global warming to 2°C above preindustrial levels (Pachauri et al., 2014). Solutions to minimize the cost of the environmental deterioration caused by rapid economic growth are encouraged at the macro-level (e.g., Tian et al., 2020; Wan et al., 2021). To combat global warming, over recent decades, many green technologies, such as wooden computer chips, wireless mice, innovative lightbulb designs, and plastic roads have been introduced (Wang et al., 2021). However, recent research (e.g., Gillingham et al., 2013, 2016) suggests that increased attention to green technological innovations as a way to adapt to climate change might undermine people's, particularly university students' perceived climate change risk and pro-environmental behavior intentions since university students are the group that is most receptive to inventions. Irrational responses to green tech innovation can be referred to as risk compensation behaviors. That is, learning about new techniques may, in fact, reduce concerns about global warming due to the belief that climate change can be managed to some degree through these new green techniques (Bingswanger, 2001; Herring, 2006).

The advocation of new green technologies may cause individuals to feel morally “off the hook” (Zhong et al., 2009) or that the problem has been dealt with, reducing the likelihood of adopting other pro-environmental actions (Weber, 1997). For example, Weber (1997) found that American farmers who had adapted their production practices in response to climate change (e.g., through crop selection or tillage practices) were less likely to adopt a price-based adaptation action (e.g., using futures contracts) and were less supportive of government interventions to curb climate change. Thøgersen and Crompton (2009) found that the implementation of a new biodegradable plastic bag not only increased Danish consumers' plastic bag consumption but also reduced their other recycling behaviors. Multiple studies on safety and health behaviors have also found a relationship between the way a remedy is advertised and its impact on risk compensation behaviors (Dilley et al., 1997; Kelly et al., 1998; Bolton et al., 2006).

In recent years, there has been a growing interest in research to reduce the risk compensation effect related to the negative spillover effects of mentioning climate change adaptation technology on people's intention to adopt pro-environmental behaviors. However, the academic literature in this area is very limited, which sharply contrasts with the economic relevance of climate change issues. The aim of this study is to apply both construal level theory and message framing technique on mitigating participants' reduced climate change risk perception and pro-environmental behavior caused by the risk compensation effect. More specifically, the goal of this study is to utilize a psychological factor (i.e., mental construal level) based on construal level theory, which is widely used in social psychology, in conjunction with the message framing method to remedy people's irrational responses to new “green” technologies, or “risk compensation” behavior.

This paper presents the results of three experiments conducted on 1,500 university students in Chongqing China. The results show that highlighting a new technology as playing a promotional/preventive role related to climate change can significantly mitigate university students' risk compensation behavior so as to promote their pro-environmental behavior when they are at low/high mental construal level (i.e., for concrete/abstract thinking), respectively. We examined participants' perceived climate change risk and pro-environmental behavior using a survey before and after interventions. To the best of our knowledge, this is the first study to apply a combination of construal level theory and message framing method on the mitigation of risk compensation behavior among university students related to climate change. We also contribute to the literature that examines the effectiveness of promotional-framed and preventive-framed messages in various contexts. Previous studies have focused on smoking cessation (e.g., Toll et al., 2007; Goodall and Appiah, 2008), disease prevention behaviors (e.g., Latimer et al., 2007; O'Keefe and Jensen, 2007), and consumers' recycling intentions (e.g., White et al., 2011). Our study extends the literature by combining a message frame with construal level theory on mitigation of the risk compensation behavior in climate change.

The remainder of the paper is structured as follows: we first present the conceptual background in section conceptual background. After the presentation and discussion of our experimental design, methodology, and empirical results in section experimental design and methodology, we conclude the paper with a summary of our key findings and their policy implications in section limitations and future research.

## Conceptual Background

### Construal Level Theory

Construal level theory (Liberman and Trope, 1998; Trope and Liberman, 2010) proposes that temporal distance, defined as the perceived proximity of an event in time, changes people's responses to events by altering their mental representations of those events. The greater the temporal distance, the more likely events are to be represented in abstract, general, and decontextualized terms that convey the perceived essence of the events (high-level construals) rather than in more concrete, contextual, and incidental terms related to the events (low-level construals). To illustrate, a person thinking about a conference a year from now might think about it in terms of more superordinate goals, such as “learning about new research,” whereas a person thinking about a conference that takes place tomorrow might be construing it in terms of more subordinate and concrete goals, such as “ironing one's pants.” Researchers have suggested that high-level construals are abstract, general, structured, superordinate, and decontextualized and that low-level construals are concrete, unstructured, subordinate, incidental, and contextual (Liberman and Trope, 1998; Trope and Liberman, 2010; Wang et al., 2019).

High and low construal level thinking are both used naturally by people when thinking about objects or behavior (Wakslak et al., 2007). In addition to the fact that people can shift between high and low levels of construal, the construal level can be experimentally manipulated by the message framing (Freitas et al., 2004).

### Message Framing

Message framing is one of the most commonly manipulated features influencing people's attitudes and behaviors (Maheswaran and Meyers-Levy, 1990; Shan et al., 2020). Previous research shows that types of framing, for example, gain/loss framing, alter individuals' attention to messages and their subsequent comprehension prior to making judgments (Meyers-Levy and Maheswaran, 2004). Researchers have examined the effectiveness of framing persuasive messages in various contexts, such as smoking cessation (e.g., Goodall and Appiah, 2008), disease prevention (O'Keefe and Jensen, 2007; e.g., Latimer et al., 2007), and consumer recycling (e.g., White et al., 2011). Messages can also focus on either the immediate benefits of pursuing an action (i.e., “preventive frame”) or the more temporal distance (longer-term) benefits of pursuing an action (i.e., “promotional frame”).

For example:

**The promotional frame of green tech innovation:**

*The new green technology can make air conditioners run stably without increasing energy consumption in an environment of global temperature rise. This technology will enable people to address global warming better*.

**The preventive frame of green tech innovation:**

*The new green technology can significantly reduce air conditioner energy consumption and greenhouse gas emissions, thereby reducing the threat of global warming to human beings*.

Individuals react differently to objectively identical information depending on the message framing, on whether an immediate benefit (i.e., preventive frame) or a more temporal distance benefit (i.e., promotional frame) is highlighted.

### Interaction Between Construal Level and Message Framing

Lee and Aaker (2004) and Thompson and Hamilton (2006) demonstrated the congruency effect between message orientation and an individual's construal level in various contexts. For instance, Lee and Aaker (2004) found that participants' information processing is facilitated when the message frame is compatible with their construal level. This sense of “feeling right” mediates the persuasive effect. Since preventively framed messages focus on the more immediate benefits of a technology or pursuing action and promotionally framed messages focus on more temporal distance benefits of a technology or of pursuing an action, it is anticipated that a preventively framed message will be particularly effective when paired with a low construal mindset focusing on temporal distance, whereas a promotionally framed message will be more effective when matched with a mindset that engages higher-level, more temporal distance thinking. In other words, a match between preventively/promotionally framed information and a low/high construal mindset leads to enhanced fluency or ease of understanding and processing the information, which subsequently will make the information more persuasive. Therefore, when individuals form an abstract thought (i.e., high construal level), learning of a green technological innovation that is framed as playing a preventive (low construal level) role related to climate change can mitigate their risk compensation behavior due to the belief of abstract thinkers that the preventive action is not an efficient “solution” to global warming. This high and low combination results in an incongruent intervention that is not persuasive enough to make participants believe that the new technology is an efficient remedy for climate change. Taken together, the mismatch between an individual's construal level and new technology message framing will eventually mitigate their risk compensation behavior.

## Experimental Design and Methodology

To mitigate risk compensation behavior, we propose that when individuals are in a low construal mindset, learning about a green technological innovation that is framed as playing a promotional role related to climate change can mitigate their risk compensation behavior, due to the belief of abstract thinkers that the preventive action is not an immediate “solution” to climate change, whereas when individuals are in a high construal mindset, learning of a green technological innovation that is framed as playing a preventive role related to climate change can also reduce their risk compensation behavior, due to the belief of abstract thinkers that the preventive action is only a partial and temporary “solution” to climate change. Overall, the incongruent combination of message framing and construal level results in less persuasive messaging and makes participants believe that the conveyed technology is not an efficient remedy for climate change. As a result, this leads to less risk compensation behavior.

### Experiment One

Experiment one tests for the predicted interaction between mental construal (abstract vs. concrete) and the type of benefit (i.e., preventive vs. promotional) that is highlighted when advocating the effect of a new “green” technology on the shift of public climate change risk perception.

***H1a:*** *Advocating “green” technological innovation undermines climate change risk perception due to the feeling of being assured*.
***H1b:*** *The response to “green” technological innovation (i.e., reduced climate risk perception) varies with individual construal level (or mental mindset)*.
***H1c****: When individuals form an abstract thought (i.e., high construal level), learning of a green technological innovation that is framed as playing a preventive (i.e., mitigation) role related to climate change can mitigate their reduced climate change risk perception (risk compensation behavior) due to the belief of abstract thinkers that preventive action is only a partial and temporary “solution” to climate change and that the new technology is less likely to mitigate climate change risk*.
***H1d:*** *When individuals form a concrete thought (i.e., low construal level), learning of a green technological innovation that is framed as playing a promotional (i.e., adaption) role related to climate change can mitigate the reduced climate change risk perception (risk compensation behavior) due to the belief of concrete thinkers that the promotional action is not an immediate “solution” to climate change and the technology is not an efficient way to solve the climate change issues*.

#### Experiment Design

**Figure 1:**
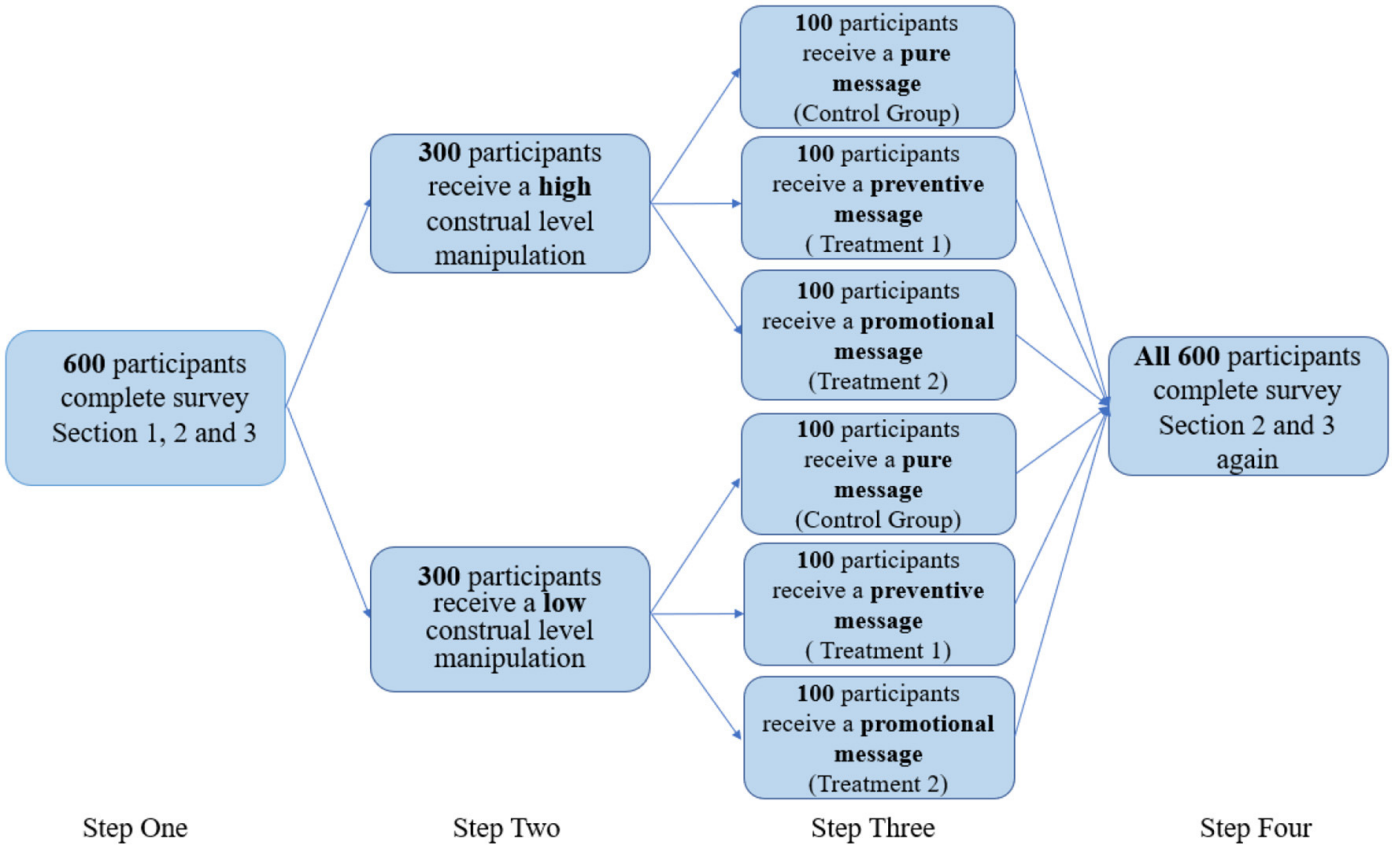
Experimental design.

The experiment was a 2 (high construal level vs. low construal level) × 3 (non vs. mitigation vs. adaptation) mixed design (see Figure 1, where the outcomes are changes in climate risk perception). It involves an online survey in which participants complete 15–20 min pre-and posttest surveys and read three articles describing new “green” technologies between the two surveys. The mental construal level was manipulated using a well-established task in which participants wrote about either their life “one year from tomorrow” (abstract thinking) or their life “tomorrow” (concrete thinking) (Förster et al., 2004). In this experiment, 600 university students were recruited from an online survey platform www.wenjuan.com to follow the procedure as follows:

- **Step 1:** In the pretest survey, the participants answered a few basic demographic questions and questions from the survey on climate change risk perception and pro-environmental behavior (see Sections introduction, conceptual background, and experimental design and methodology of the survey in Appendix A) to test their baseline level of climate change risk perception and pro-environmental behavior.
- **Step 2:** Then, the participants were randomly assigned to one of two groups: (1) a group that was asked to complete a task (e.g., write three things you want to do in a year) that manipulated their mental representations into high-level construals; (2) a group that was asked to complete a task (e.g., write three things you want to do tomorrow) that manipulated their mental representations into low-level construals.
- **Step 3:** Then, the participants randomly received one of three versions of the articles (see Appendix B), in which the new green technology varied from pure technology (control group), a technology that prevents climate change (treatment 1), and a technology that enables adapting to climate change (treatment 2).
- **Step 4:** The participants were asked to complete section conceptual background and section experimental design and methodology of the survey (see Appendix A) again to test their level of climate change risk perception and pro-environmental behavior after learning of the new “green” technologies. They were also asked to answer two questions using a 1 (strongly disagree)-5 (strongly agree) scale measuring their perceived efficiency of these new technologies related to climate change and their degree of feeling assured: (1) Do you think the technology mentioned by the article is an efficient remedy for climate change? (2) Are you feeling assured regarding climate change after learning of these “green” technologies?

#### Experiment Results

We conducted both ANOVA and regression analysis to investigate the interaction effect of mental construal and the messaging type used to advocate the new “green” technologies on the shift of public climate risk perception. We calculated the overall score of the pro-and post-reading tests on climate change risk perception for each participant and used the score difference between the two tests as a measure of perception shifting (post-intervention perceived climate change risk minus preintervention perceived climate change risk), which is the dependent variable. ANOVA was used to test for the significance of differences among the group means in our sample, and regression analysis showed the direction (e.g., larger or smaller) and magnitude (i.e., size) of these differences.

Table 1 panels A and B report the within-group mean of the climate risk perception change and the feeling of being assured. On average, both the high and low construal level groups exhibit reduced climate risk perception regardless of whether the technologies were presented as pure technologies (with noting said on climate change) or as technologies that play a preventive (i.e., mitigating) or promotional (i.e., adapting) role related to climate change. The result supports hypothesis ***H1a*** that learning of a “green” technical innovation can undermine climate risk perception and provides suggestions for a risk compensation effect. For those with high-level construals (i.e., abstract thinking), preventive messaging advocating for technology by highlighting the mitigating benefit of the technology on climate change results in the lowest feeling of assurance (i.e., *M* = 2.65, vs. *M* = 3.41 in the pure technology message (control) group, and *M* = 5.01 in the promotional message group) and thus moderates the reduced risk perception (i.e., *M* = −1.42 vs. *M* = −2.18 in the pure technology message (control) group and *M* = −3.78 in the promotional message group). This is because the combination of a high construal mindset and a preventive frame is an incongruent intervention. This combination is not effective in persuading participants to believe that the new technology is able to mitigate the climate change risk.

**Table 1:** Within-group mean of dependent variables.

**(A) Change in risk perception (Experiment 1)**			
	**High construal**	**Low construal**	**Total**
Pure (control)	−2.18	−2.62	−2.42
Preventive message (T1)	−1.42	−4.50	−2.79
Promotional message (T2)	−3.78	−1.02	−2.23
Total	−2.86	−1.96	−2.38
**(B) Feeling of being assured (Experiment 1)**			
	**High construal**	**Low construal**	**Total**
Pure (control)	3.41	3.85	3.65
Preventive message (T1)	2.65	5.73	4.02
Promotional message (T2)	5.01	2.25	3.46
Total	4.09	3.19	3.61
**(C) Pro-environmental behavioral intention (Experiment 2)**			
	**High construal**	**Low construal**	**Total**
Pure (control)	−1.29	−1.73	−1.40
Preventive message (T1)	0.04	−3.47	−1.52
Promotional message (T2)	−2.01	−0.50	−1.17
Total	−1.42	−0.95	−1.25
**(D) Actual pro-environmental behavior (Experiment 3)**			
	**High construal**	**Low construal**	**Total**
Pure (control)	8.91	9.35	9.15
Preventive message (T1)	8.15	11.23	9.52
Promotional message (T2)	10.51	7.75	8.96
Total	9.59	8.69	9.11

*(A) Reports the within-group mean of the difference in climate risk perception before and after the intervention. (B) Reports the within-group mean of the feeling of being assured. (C) Reports the within-group mean of the change in pro-environmental behavioral intentions. (D) Reports the within-group mean of the actual pro-environment behavior*.

Likewise, those with low-level construals (i.e., concrete thinking) were the least assured by promotional messages highlighting the climate-change adaptation (long term) benefit of the technologies. Again, this combination results in an incongruent intervention that is not persuasive enough to make participants believe that the technology is an efficient remedy for climate change. Consequently, this leads to less reduction in risk perception (*M* = −1.02 vs. *M* = 2.62 in the pure technology message (control) group and *M* = −4.50 in the preventive message group). Overall, the low construal group presented promotional messages advocating green technologies experience a decreased compensation effect and thus exhibit a less reduced risk perception. These findings provide supportive evidence for hypotheses *H1b, H1c*, and *H1d*.

Table 2 panel A presents the results of both ANOVAs. The results of the ANOVA indicated a significant interaction between mental construal and highlighting preventive (i.e., mitigating) vs. promotional (adapting) benefits of green technology on changes in climate risk perception (see Interaction effect in Table 2), where [*F*_(2, 300)_ = 9.85, *p* = 0.0001], and F is the F statistic. In contrast, the main effects of construal level and message type were not significant (i.e. [*F*_(1, 300)_ = 0.20, *p* = 0.6533; and *F*_(2, 300)_ = 0.33, *p* = 0.7171]. To further corroborate the interaction effect, we checked the simple main effect of message type by mental construal level and the simple main effect of construal level for the message type. All *p*-values [see Panel A (a)–(d)], except those for preventative messaging, indicate significance.

**Table 2:** Testing for the effects of mental construal, technology messaging type, and their interaction on the response.

**DV: change in risk perception**			
**(A) ANOVA**			
	***F*** **test stats**	***P***	***df***
Main effect: Mental construal level	0.20	0.6533	1
Main effect: Type of message	0.33	0.7171	2
Interaction effect: Mental construal level* Type of message	9.85	0.0001	2
Simple main effect (High construal): Type of message	6.44	0.0016	2
Simple main effect (Low construal): Type of message	5.25	0.0026	2
Simple main effect (Preventive): Construal level	5.92	0.0149	1
Simple main effect (Promotional): Construal level	16.66	0.0000	1
**(B) Regression analysis**			
	**High construal**	**Low construal**	
Preventive message	1.084 (0.211)^***^	–1.846 (0.838)^***^	
Promotional message	−0.927 (1.141)	1.921 (0.947)^***^	
Constant	−1.199 (0.187)^***^	−2.297 (1.145)^***^	
*N*	300	300	
Adj R-squared	0.153	0.144	
Including control variables	Yes	Yes	

*(A,B) Present the ANOVA and regression results. The dependent variable is the difference in climate risk perception before and after the intervention*.

*Standard errors are in parenthesis*.

*** *Significant at 1%*.

**Figure 2:**
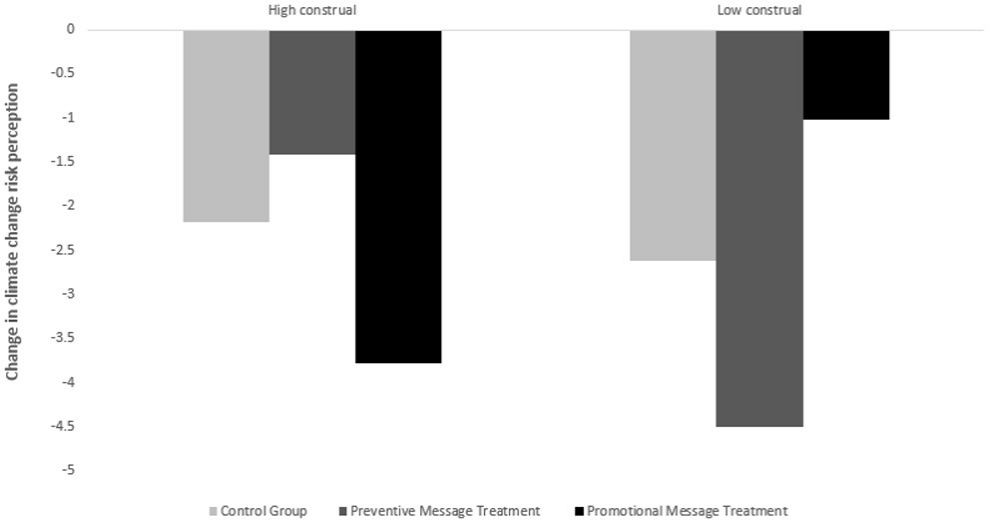
Change in pro-environmental behavioral intention.

Next, we examine the regression results in Table 2 panel B. The dependent variables for both regressions are the difference in participants' perceived climate change risk (preintervention perceived climate change minus post-intervention perceived climate change). We separately run the regressions for the high and low construal level groups, and the results clearly indicate an interaction effect between mental construal level and the message type used to advocate the technologies. We include two dummy variables as independent variables to indicate the preventive and promotional message groups, and we include some demographic variables, such as age and sex, as controls. Looking at the column of “high construal,” the high construal level group presented preventive messages exhibited significantly less reduced climate risk perception [i.e., coefficient = 1.084 and s.d. (coefficient) = 0.021] compared with the benchmark group presented pure technology messages. Comparatively, the promotional message group showed more reduced risk perception than the benchmark group, but the difference is insignificant. Moving toward the “Low construal level” column, the opposite effect is seen. The reduced risk perception in the promotional message group decreased by 1.921 points, whereas the reduced risk perception in the preventive message group increased by 1.846 points. These findings are further supported by Figure 2, which shows bar graphs that compare the change in participants' climate change risk perception before and after the experimental intervention.

Taken together, we confirm the interaction effect of mental construal level and the way “green” technology is advocated on the shift of public perceived climate risk. In particular, the lack of fit between a mental representation and a message advocating technology as solutions to climate change helps remedy the decrease in climate risk perception. Meanwhile, the results reported in Table 3 indicate that the change in risk perception is due to the feeling of being assured by the message introducing the technological innovations.

**Table 3:** Testing for the effects of mental construal, technology messaging type, and their interaction on a response (Experiment 1: Feeling of assured).

**DV: Feeling of being assured**			
**(A) ANOVA**			
	***F* test stats**	***P***	***df***
Main effect: Mental construal level	0.20	0.6533	1
Main effect: Type of message	0.33	0.7171	2
Interaction effect: Mental construal level* Type of message	9.85	0.0001	2
Simple main effect (High construal): Type of message	3.63	0.0265	2
Simple main effect (Low construal): Type of message	6.44	0.0016	2
Simple main effect (Preventive): Construal level	5.92	0.015	1
Simple main effect (Promotional): Construal level	16.67	0.0000	1
**(B) Regression analysis**			
	**High construal**	**Low construal**	
Preventive message	−1.084 (0.021)^***^	0.264 (0.075)^***^	
Promotional message	0.927 (1.141)	−0.482 (0.06)^***^	
Constant	2.429 (1.184)^***^	2.861(0.798)^***^	
N	300	300	
Adj R-squared	0.135	0.156	
Including control variables	Yes	Yes	

*(A,B) Present the ANOVA and regression results. The dependent variable is the feeling of being assured. Standard errors are in parenthesis*.

*** *significant at 1%*.

### Experiment Two

We demonstrated in experiment one that a mismatch between a mental representation and a message framing could effectively attenuate people's lowered climate change concerns caused by risk compensatory behavior. However, it is unclear if greater climate change risk perception would translate into increased individual action to preserve the environment. Consequently, we ran experiment two to examine whether people's heightened concern about climate change would lead to more pro-environmental action intentions.

*H2a: Changes in climate risk perception can transfer to pro-environmental behavioral intentions*.

*H2b: When individuals form an abstract thought (i.e., high construal level), learning about a green technological innovation that is framed as playing a preventive (i.e., mitigation) role related to climate change can attenuate the reduced pro-environment behavioral intentions caused by the alleviation of lowered climate change risk perception*.

*H2b: When individuals form a concrete thought (i.e., low construal level), learning about a green technological innovation that is framed as playing a promotional (i.e., adaption) role related to climate change can attenuate the reduced pro-environment behavioral intentions caused by the alleviation of lowered climate change risk perception*.

#### Experiment Design

In this experiment, another 600 university students were recruited from the online platform www.wenjuan.com. The experimental procedure was the same as that described in Experiment 2, but two differences were presented. First, for the robustness test, we use an alternative way of manipulating participants' mental construal levels. The participants were presented with 10 tasks and asked to indicate either “why” we need to complete the tasks (to manipulate their mental representations to form a high construal) or “how” we can complete the tasks (to manipulate their mental representations to form a low construal) Förster et al. (2004). Second, we used pre-and post-tests to measure the participants' pro-environmental behavioral intentions using the survey questions shown in Appendix A section experimental design and methodology.

#### Experiment Results

Table 1 panel C reports the within-group mean of the change in pro-environmental behavioral intentions. The change is measured by the difference in the pro-environmental behavior intention scores before and after reading the articles. On average, both the high- and low-construal-level groups exhibited reduced intentions, regardless of the type of message they received. The results support hypothesis H2a that pro-environmental behaviors can be impacted by changes in climate risk perception. Similar to what we observed in Experiment 1, the participants with high-level mental construals (i.e., abstract thinking) showed the least reduced pro-environmental behavioral intentions when presented preventive messages about technologies with a mitigating benefit on climate change. For those with low-level mental construals (i.e., concrete thinking), using promotional messages that highlight the adaptation benefits of technologies on climate change had the largest moderating effect on the reduced pro-environmental behavioral intentions. These findings provide supportive evidence for hypotheses H2b and H2c.

**Figure 3:**
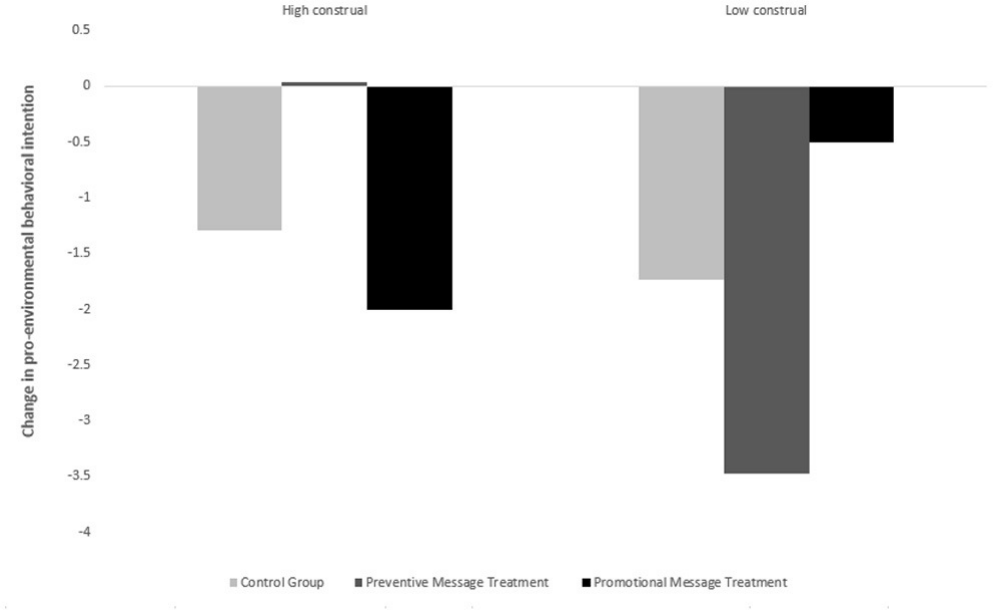
Change in climate change risk perception.

Table 4 panel A presents the ANOVA and regression results. The results of the ANOVA indicated a significant interaction effect between mental construal and messages highlighting preventive (i.e., mitigating) vs. promotional (adapting) benefits of a technology related to climate change on pro-environmental behavior intentions. The *F* test stat of the interaction effect is 9.98 with a *p* = 0.0001, which is significant even at the 1% level. All the *p*-values for the simple main effects, except for those for the effect of message type on those with high construals, were significant [see Panel A (a)-(d)]. The regression results are presented in Panel B. Once again, we observe that learning of “green” technologies reduces people's pro-environmental behavioral intentions (i.e., the “constants” in both regressions are significantly negative) but highlighting mitigating/adapting benefits for people with high/low level mental construals can significantly correct the reduction by 0.764 and 1.097 points, respectively. Figure 3, which depicts bar graphs comparing the change in pro-environmental behavioral intention before and after the experimental intervention, corroborates these findings.

**Table 4:** Testing for the effects of mental construal, technology type, and their interaction on response (Experiment 2: Change of pro-environment behavioral intentions).

**DV: Change in pro-environment behavioral intentions**			
**(A) ANOVA**			
	***F* test stats**	***P***	***df***
Main effect: Mental construal level	1.19	0.2762	1
Main effect: Type of message	0.67	0.5116	2
Interaction effect: Mental construal level* Type of message	9.98	0.0001	2
Simple main effect (High construal): Type of message	3.58	0.0278	2
Simple main effect (Low construal): Type of message	6.73	0.0012	2
Simple main effect (Preventive): Construal level	12.53	0.0004	1
Simple main effect (Promotional): Construal level	8.12	0.0044	1
**(B) Regression analysis**			
	**High construal**	**Low construal**	
Preventive message	0.764 (0.062)^***^	–1.057 (0.711)^***^	
Promotional message	−0.140 (0.896)	1.097 (0.432)^***^	
Constant	–0.199 (0.086)^***^	–0.145 (0.084)^***^	
*N*	300	300	
Adj R-squared	0.175	0.183	

*(A,B) Present the ANOVA and regression results. The dependent variable is the difference in pro-environmental behavioral intentions before and after the intervention. Standard errors are in parenthesis*.

*** *significant at 1%*.

Taken together, we demonstrate in experiment 2 that individuals' increased climate change concern results in increased pro-environmental action intentions which is consistent with our hypothesis.

### Experiment Three

One inherent challenge associated with surveys eliciting information about pro-environmental behavior is that they are not externally verifiable. Student participants may overstate or fabricate their actual pro-environmental behavior in the survey (Bu and Liao, 2021). To address these concerns, in experiment three, we conducted an incentivized consequential choice experiment. The basic design of the experiment allows the estimation of how participants trade-off between their private monetary earnings and environmental damage (reduction in a donation to a tree planting charity). The money-incentivized task has the advantage of reflecting participants' real intention to engage in pro-environmental activities. Experiment three tests our prediction that a shift in perceived climate change risk would change people's actual pro-environment behavior.

*H3a: When individuals form an abstract thought (i.e., high construal level), learning of a green technological innovation that is framed as playing a preventive (i.e., mitigation) role related to climate change can mitigate the reduced actual pro-environment behavior due to the belief of abstract thinkers that the preventive action is only a partial and temporary “solution” to climate change*.

*H3b: When individuals form a concrete thought (i.e., low construal level), learning of a green technological innovation that is framed as playing a promotion (i.e., adaption) role related to climate change can mitigate the reduced actual pro-environment behavior due to the belief of concrete thinkers that the promotional action is not an immediate “solution” to climate change*.

#### Experiment Design

**Figure 4:**
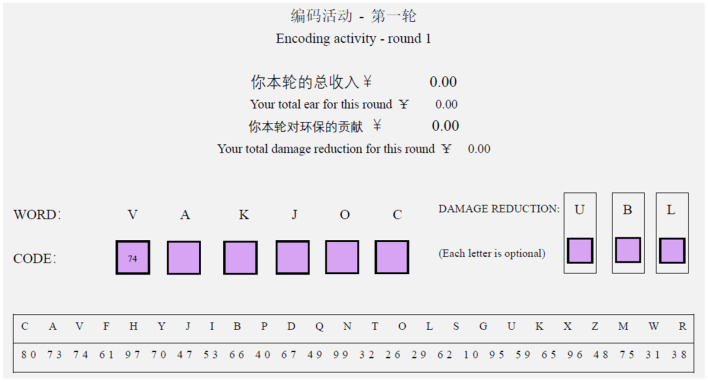
Experimental paper of the main activity.

In this experiment, 300 students were recruited from a university in Chongqing, China. The student participants completed the same manipulation of mental construal level as described in Experiment 1 and were then randomly given one of the three versions of the articles. Then, they were asked to play a word decoding effort game. The word game is based on a word decoding effort task, comparable to Dorner (2019), Erkal et al. (2011), and Benndorf et al. (2019). Each participant played three 6-min rounds word game. At the start of each 6-min round, participants are given a paper depicted in Figure 4. Participants must properly input the two-digit codes for each of the random letters in the six-letter “word” they are given. The codes are shown in a jumbled alphabet at the bottom of the paper. The word is shown in the upper left corner of the paper, as shown in Figure 4. Once a participant has properly finished the word, she could receive 1 RMB for that word. To maximize her personal earnings for the round, the player must attempt to finish as many six-letter words as possible within the 6-min time restriction.

Each completed word lowers the amount of money donated to charity for that round. The initial donation for any given round was set at 15 RMB per participant. The charity is a local tree planting organization, and participants are aware that every two RMB given to the organization results in the planting of one seedling. Additionally, participants are aware that each completed word lowers the charity contribution by 2 RMB, which is deducted from the donation of 15 RMB. However, participants may minimize the negative impact of that word on the charity by adding additional letters in the middle right of the paper. The instructions make it obvious to participants that these extra letters are optional. One additional letter reduces the damage to the charity by one-third, two additional letters reduce the damage to the charity by two-thirds, and all three additional letters reduce the damage to the charity to nothing. As a result, participants must choose a trade-off between their private profits and the amount of harm they are prepared to inflict to the charity payout in each round. The current round's cumulative earnings and damage are displayed in the top center of the paper. At the conclusion of the experiment, students were compensated for the amount earned during the activity. The money saved by the participants was then donated to a tree planting charity. The money-incentivized task has the advantage of reflecting participants' real intention to engage in pro-environmental activities.

We administered the word game to the participants via paperwork, and one round of a sample game is shown in Appendix C. The dependent variable investigated in this experiment is the averaged reduced donation amount over three rounds for each participant. For a participant, the lower the donation amount reduced, the greater the mitigation of her diminished pro-environmental behavior.

#### Experiment Results

**Figure 5:**
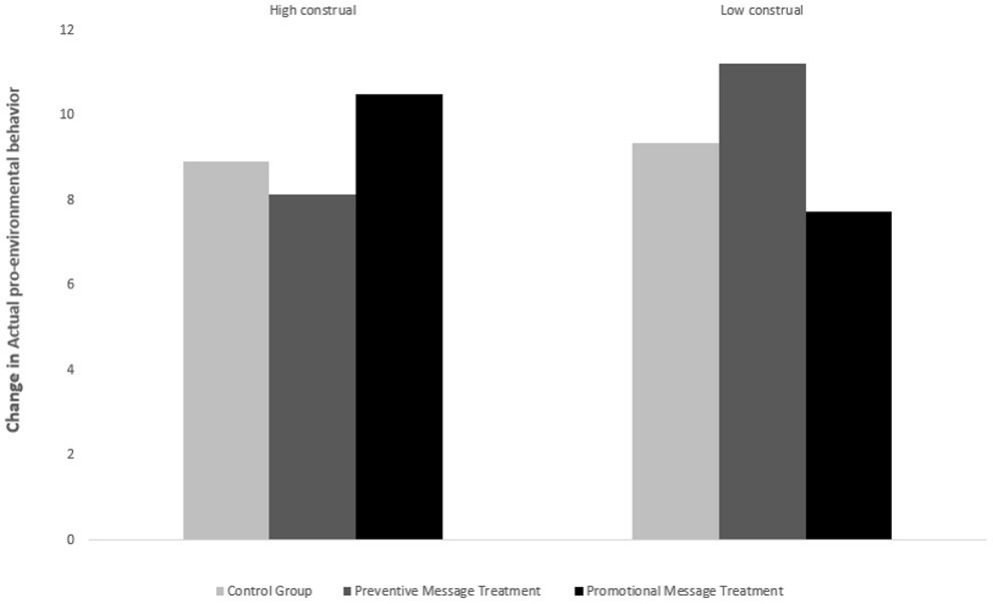
Change in actual pro-environmental behavior.

Table 1 panel D shows the within-group mean of the actual pro-environmental behavior, measured by the difference in the donation amount and the initial 15 RMB donations. The higher the value is, the less the participant donated to the charity. Similarly, the participants with high/low-level construals (i.e., abstract thinking) reduced the charitable donation the least when presented with preventive/promotional messages about technologies that play a mitigating role related to climate change. This supports hypotheses H3a and H3b. Table 5 Panel A presents the ANOVA and regression results. The results of the ANOVA still indicate a significant interaction effect between mental construal and messages highlighting the preventive (i.e., mitigating) vs. promotional (adapting) benefits of technology for climate change on actual pro-environment behavior. The F test stat of the interaction effect was 9.85 with a *p* = 0.0001. All the *p*-values for simple main effects, except for those of the effect of message type on people with high construal levels and the effect of construal level when using preventive messages [see Panels A (a)-(d)] on advocating “green” technologies, were significant. The regression results are presented in Panel B. In addition to the demographic characteristics, we also include the productivity indicator we obtained from the practical test to control for the intrinsic word processing ability of the participants. We observe that, on average, presenting preventive/promotional messages about technologies that have mitigating/adapting benefits to people with high/low construals can significantly moderate the donation deduction by 1.084 and 1.921 yuan, respectively. The results are consistent with the hypothesis. Figure 5, which provides bar graphs comparing the change in participants' actual pro-environmental behavior before and after the experimental intervention, further supports these findings.

**Table 5:** Testing for the effects of mental construal, technology type, and their interaction on the response (Experiment 3: Actual pro-environmental behavior).

**DV: Actual pro-environment behavior**			
**(A) ANOVA**			
	***F* test stats**	***P***	***df***
Main effect: Mental construal level	0.20	0.6533	1
Main effect: Type of message	0.33	0.7171	2
Interaction effect: Mental construal level* Type of message	9.85	0.0001	2
Simple main effect (High construal): Type of message	3.63	0.0266	2
Simple main effect (Low construal): Type of message	6.44	0.0016	2
Simple main effect (Preventive): Construal level	5.92	0.015	1
Simple main effect (Promotional): Construal level	16.67	0.000	1
**(B) Regression analysis**			
	**High construal**	**Low construal**	
Preventive message	−1.084 (0.211)^***^	1.846 (0.839)^***^	
Promotional message	0.927 (0.143)^***^	-1.921 (0.948)^***^	
Constant	7.929 (1.184)^***^	9.027 (1.145)^***^	
N	300	300	
Adj R-squared	1.454	1.563	

*(A,B) Present the ANOVA and regression results. The dependent variable is the difference in actual pro-environmental behavior before and after the intervention. Standard errors are in parenthesis*.

*** *significant at 1%*.

## Limitations and Future Research

The study's design has several advantages, but it also comes with some drawbacks. To begin, we conducted our research on a group of university students, many of whom were living on their own for the first time. This may have influenced their susceptibility to interventions, as people are more inclined to change their habits following a move. This begs the question of whether our intervention's effects can be generalized to more stable household circumstances as well. Nonetheless, this group may be particularly interesting because they represent future environmentalists. Future research should examine the effectiveness of a similar intervention in diverse settings, such as a factory or a household.

Another limitation is that participants were aware that they were being monitored throughout the trial in our study. This, of course, could have an impact on their behavior. However, because individuals in different treatment groups received identical information but not interventions, we could account for potential effects associated with the concept of monitoring. Although we randomly assigned participants into different treatment groups to account for potential monitoring effects, it would be interesting to observe if comparable results occur when individuals are unaware they are being monitored.

The role of peer effects in decision making has been largely explored in many contexts, such as green product adoption, saving and borrowing decisions (Georgarakos et al., 2014; Agarwal et al., 2016; Bu et al., 2021). It is well-documented in those studies that people can learn from their friends' or colleagues' experiences, and can be influenced by their choices (Hirshleifer, 2020). While peer impacts are believed to influence individuals' perceptions of climate change danger and pro-environmental action, little study has been conducted thus far. Additional research should be performed to ascertain whether and how an individual's enhanced pro-environmental behavior affects peers. Moreover, for future research, machine learning techniques could be used to assess treatment effects in this type of trial.

## Discussion and Conclusions

The literature shows that increased attention to climate change adaptation, especially green technology innovation, reduces individuals' intentions, particularly university students' intentions to mitigate global warming. The risk compensation hypothesis suggests that remedies to reduce the impacts of risky behaviors can unintentionally increase pro-environmental behaviors. However, the need for measures to adapt to climate change, which typically involves making technological changes to cope with the impacts of climate change, in addition to efforts to reduce greenhouse gas emissions, has been widely acknowledged by scientists and policymakers. In this context, reducing risk compensation behavior is important for both climate mitigation and adaptation.

In this context, this study combines a psychological factor (i.e., mental construal level) with a targeted messaging method to alter university students' risk compensation behavior. Specifically, we conducted three experiments on mitigating risk compensation behavior in the setting of climate change risk perception and pro-environmental behavior with 1,500 university students from both an online survey platform and a university in Chongqing, China, in 2019. The sample size in our study is substantially greater than comparable studies. For example, Wang et al. (2019) tested whether construal level and psychological distance from climate change predicted pro-environmental intentions with 752 subjects in their experiments. Wu et al. (2017) investigated whether construing morality at a high vs. a low level causes greater self-control with only 192 university students, whereas Griffioen et al. (2016) recruited 197 students for an experiment to determine which construal level combinations result in the most successful interventions. The relatively larger sample size allows us to estimate the treatment effect with a desired statistical power.

The results demonstrate that highlighting a new technology as playing a preventive/promotional (i.e., mitigation/adaption) role related to climate change can mitigate the risk compensation behavior of individuals with high/low mental construals (i.e., showing abstract/concrete thinking). We provide evidence that this mitigation is driven by the lack of fit between the participants' abstract/concrete thinking and the immediate/long-term solution to climate change. The incongruent combination makes messages less persuasive and allows participants to believe that the technology is not an efficient remedy for climate change. Consequently, this significantly promotes university students' pro-environmental behavior.

This paper also has implications for policymakers. The need for measures to address climate change (which typically involves green technological innovation to cope with the impacts of climate change) and efforts to reduce greenhouse gas emissions have been widely acknowledged by scientists and policymakers (IPCC, 2007; National Research Council, 2010; Keskitalo, 2012). However, learning about measures to address climate change has been found to undermine people's support for climate change mitigation. It is therefore important to develop effective measures to reduce the negative spillover effects of green technological innovation on pro-environmental actions. Our study proposes customized psychological interventions for people by construal level theory and effectively reduces the negative spillover effect.

## Data Availability Statement

The raw data supporting the conclusions of this article will be made available by the authors, without undue reservation.

## Ethics Statement

The studies involving human participants were reviewed and approved by School of Finance, Chongqing Technology and Business University. The patients/participants provided their written informed consent to participate in this study.

## Author Contributions

All authors listed have made a substantial, direct and intellectual contribution to the work, and approved it for publication.

## Funding

This research is supported by The National Social Science Fund of China: Research on Mechanism Innovation and Effect Evaluation of Ecological Poverty Alleviation from the Perspective of Targeted Poverty Alleviation (17BJY131).

## Conflict of Interest

The authors declare that the research was conducted in the absence of any commercial or financial relationships that could be construed as a potential conflict of interest.

## Publisher's Note

All claims expressed in this article are solely those of the authors and do not necessarily represent those of their affiliated organizations, or those of the publisher, the editors and the reviewers. Any product that may be evaluated in this article, or claim that may be made by its manufacturer, is not guaranteed or endorsed by the publisher.
