# Supplementary material for: The Roles of Mental Construal Level Theory in the Promotion of University Students' Pro-environmental Behaviors
Source: Front Psychol. 2021 Oct 28;12:735837. doi: 10.3389/fpsyg.2021.735837 (PMC8581237; doi:10.3389/fpsyg.2021.735837)
Supplement: Supplementary file 1 [file Data_Sheet_1.pdf]

Appendix A Survey questions

Section 1: Demographic information

- What is your age?
- What is your sex?
  1=Male
  0=Female
- What is your marital status?
  1=Married
  0=Single
- What is your education level?
  1 = no schooling;
  2= primary school;
  3 = junior high school;
  4= senior high school
  5 = Bachelor's or above.

Section 2: Perceived climate change risk

We created 18 items measured on a common response scale ranging from 1 (strongly disagree) to 5 (strongly agree).

- How concerned are you about global warming?
- How likely do you think it is that each of the following will occur during the next

50 years due to global warming?

- Worldwide, many people’s standard of living will decrease
- Worldwide water shortages will occur.
- Rates of serious diseases will increase worldwide.
- My standard of living will decrease.
- Water shortages will occur where I live.
- My chance of getting a serious disease will increase.
- How serious of a threat do you believe global warming is to nature?
- How serious are the current impacts of global warming around the world?

Section 3: Pro-environmental behavior

Please state the extent to which you agree with the following statements (1 = strongly disagree, 7 = strongly agree)

- I wait until I have a full load before I wash my clothes in the washing machine to save water
- I wash my clothes at a lower temperature to save energy
- I keep the pressure/flow of the shower at a rate lower than what I consider to be ideal for saving water
- I limit the time I spend in the shower to reduce my water consumption
- When I am done charging an appliance, I take the charger out of the socket
- When I boil water, I boil only as much as I need
- When there is nobody in a room, I switch off the light
- I keep the shower at a temperature lower than what I consider to be an ideal temperature to save energy
- I turn off the shower when I am soaping myself down
- I switch appliances off instead of leaving them on standby
- How often… (1 = never, 5 = always)
- Are the products you buy organic?
- Do you eat meat?
- Do you bring glass bottles to the recycle bin?

- Do you separate your paper from your waste?
- Do you separate your waste (chemical, plastics, organic)?
- Do you search for environmentally friendly products?

Appendix B Sample articles

The three articles attached below are the ones the participants read during the experiments. Each article has three versions with (pure (control), preventive (T1) vs. promotional (T2)) messages and is written in Chinese.

1. New Hydrocarbon Refrigerant HCR22 for Air Conditioning (Pure)

In hot weather, the air conditioning (AC) of a building consumes a large amount of energy, which exerts great pressure on the power grid. If the newly developed HCR22 hydrocarbon refrigerant is used, **it can significantly reduce the energy consumption of AC and the wear on AC systems to relieve the pressure on the power grid during summer peak use.**

The new HCR22 environmental protection hydrocarbon refrigerant uses refined high purity propane, butane, and other mixed alkanes. It is a deeply purified, precisely prepared, safe, and environmentally friendly refrigerant mixed at a certain weight ratio. The unit refrigerating capacity of HCR22 is large, and the latent heat value of vaporization is 1.84 times that of ordinary refrigerant; thus, its cooling speed is extremely fast, allowing air conditioners to reach the desired temperature quickly and shut down. The unit refrigerating capacity meets requirements and is better than that of ordinary refrigerants. After a large amount of HCR22 use, data show that using HCR22 in place of ordinary AC coolant results in a 15% power consumption reduction, **which reduces the pressure on the power grid and reduces the failure rate of the urban power grid.**

1. New Hydrocarbon Refrigerant HCR22 for Air Conditioning (Preventive)

In hot weather, the air conditioning (AC) of a building consumes a large amount of energy, **which emits substantial greenhouse gas**. If the newly developed HCR22 hydrocarbon refrigerant is used, the energy consumption of AC and the wear on AC systems can be significantly reduced to **reduce greenhouse gas emissions and alleviate global warming.**

The new HCR22 environmental protection hydrocarbon refrigerant uses refined high purity propane, butane, and other mixed alkanes. It is a deeply purified, precisely prepared, safe, and environmentally friendly refrigerant mixed at a certain weight ratio. The unit refrigerating capacity of HCR22 is large, and the latent heat value of vaporization is 1.84 times that of ordinary refrigerant; thus, its cooling speed is extremely fast, and air conditioners reach the desired temperature quickly and shut down. The unit refrigerating capacity meets requirements and is better than that of ordinary refrigerants. After a large amount of HCR22 use, data show that using HCR22 in place of ordinary AC results in a 20% energy saving rate increase, **reducing greenhouse gas emissions by 15% to reduce the threat of global warming to human beings.**

1. **New Hydrocarbon Refrigerant HCR22 for Air Conditioning (Promotional)**

In hot weather, the air conditioning (AC) of a building consumes considerable energy, exerting great pressure on the power grid. The newly developed HCR22 hydrocarbon refrigerant can significantly reduce AC energy consumption and AC system wear **to better adapt to the temperature rise caused by global warming in summer and enable AC equipment to operate normally in the case of a temperature increase.**

The new HCR22 environmental protection hydrocarbon refrigerant uses refined high purity propane, butane, and other mixed alkanes. It is a deeply purified, precisely prepared, safe, and environmentally friendly refrigerant mixed at a certain weight ratio. The unit refrigerating capacity of HCR22 is large, and the latent heat value of vaporization is 1.84 times that of ordinary refrigerant; thus, its cooling speed is extremely fast, and air conditioners reach the desired temperature quickly and shut down. The unit refrigerating capacity meets requirements and is better than that of ordinary refrigerants. A large amount of HCR22 usage data show that **after adopting**

HCR22, the power consumption of ordinary air conditioners is reduced by 15%. Given the global temperature rise, this technology can make air conditioners run stably without increasing energy consumption. This technology will enable people to adapt to global warming better.

Appendix C Sample of Word encoding game

The activity consists of a word encoding task. The task involves correctly assigning numbers to up to 6 random letters in a word that you are given. You will be given the full alphabet in random order. A number is presented below each letter. Here is the full alphabet:

D	P	Q	E	O	U	I	T	S	R	V	F	N	B	C	H	W	A	N	Y	X	Z	K	L	J
59	28	25	91	87	11	20	26	49	34	61	35	99	85	82	63	96	83	38	76	88	67	29	46	17

Now you will see six randomly selected letters. This is your “word.” Below each letter is an empty box, as shown here. Provide the code for them.

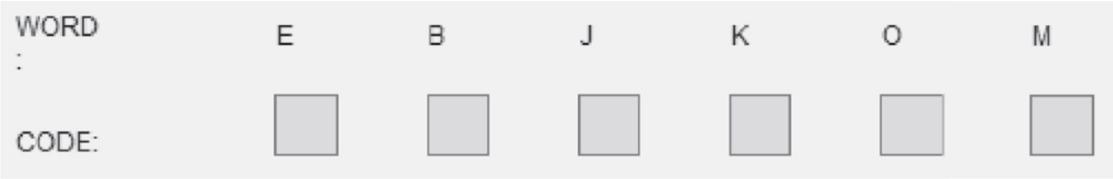

Payment: you will earn 1 yuan for each letter that is correctly coded

Deduction from charitable donation: we will give you an initial 15 yuan for a charitable donation to a tree planting society. However, a correct word (that is, correctly coding all six letters) reduces the donation amount by 2 yuan and transfers the amount to you.
